# Triple-Negative Breast Cancer: Tumor Immunogenicity and Beyond

**DOI:** 10.1155/2024/2097920

**Published:** 2024-10-04

**Authors:** Elio Ibrahim, Ernest Diab, Rony Hayek, Karim Hoyek, Hampig Kourie

**Affiliations:** ^1^Faculty of Medicine, Saint Joseph University, Beirut, Lebanon; ^2^Oncology Department, Faculty of Medicine, Saint Joseph University, Beirut, Lebanon

## Abstract

Triple-negative breast cancer (TNBC) is a breast malignancy with a poor prognosis and limited therapeutic options. Many studies show that TNBC exhibits heterogeneity across clinical, histopathological, and molecular levels. In this review, we discuss the immunogenic features of TNBC with a focus on immunotherapy and the current standard of care in the neoadjuvant, adjuvant, and metastatic setting. In addition, we address the ongoing research on immunotherapy, antibody-drug conjugates (ADCs), poly ADP-ribose polymerase (PARP) inhibitors, and future challenges in the treatment of this entity.

## 1. Introduction

Breast cancer (BC) is a prevalent malignancy with an estimated 2.3 million new cases reported globally in 2020. In the United States, BC accounts for 32% of all cancers in women as of 2024 [1]. Notably, a comparison of four simulation models reveals that BC screening and treatment in 2019 led to a 58% reduction in BC mortality in the United States when compared to the interventions employed in 1975 [2].

Many factors influence the complex epidemiology of BC such as age, genetic predisposition, lifestyle factors, hormonal influences, and environmental exposures [3]. Triple-negative BC (TNBC) represents 11%–20% of all BCs and is more prevalent in premenopausal women and individuals with mutations in the BC BRCA1/2 genes [4]. BC is classified into different subtypes according to the expression profiles of the estrogen receptor (ER), progesterone receptor (PR), and human epidermal growth factor receptor 2 (HER2). TNBC is defined by the lack of all three receptors. Because of the absence of obvious biological targets or markers, chemotherapy has been the mainstream treatment for years, with only a fraction of TNBC patients significantly benefiting from it [5].

Many studies revealed that TNBC exhibits heterogeneity across clinical and histopathological levels [6]. Understanding the molecular and genetic characteristics of TNBC is essential to classify patients and better identify molecular-based therapies. Lehmann et al. classified TNBC based on gene expression in six different subtypes: two basal-like (BL1 and BL2), an immunomodulatory (IM), a mesenchymal (M), a M stem–like (MSL), and a luminal androgen receptor (AR) (LAR) subtype. The BL1 and BL2 subtypes displayed greater expression of cell cycle and DNA damage response genes. M and MSL subtypes were associated with elevated expression of epithelial-M transition and growth factor pathways. The LAR subtype is distinguished by AR signaling [7]. Later, Burstein et al. proposed another classification omitting the IM and MSL subtypes as they mainly depended on infiltrating lymphocytes and tumor-associated stromal cells, resulting in a consensus of four intrinsically defined TNBC subtypes. This classification did not only help better understanding of TNBC on a molecular level but also had clinical implications as it correlated with prognosis [8].

Moreover, a subset of TNBC has been shown to have high tumor-infiltrating lymphocytes (TILs) similar to those observed in melanoma or lung cancer, which raised many questions about the immunogenicity of TNBC in contrast with other BC subtypes. This further opened to light the possibility of immunotherapy treatment in TNBC [9]. Having said that, clinical trials evaluating the efficacy of immunotherapy in TNBC are revealing promising results, with research in the field booming in recent years. In this review article, we are trying to address which molecular and microenvironmental factors contribute to the pronounced immunogenicity of TNBC, along with the clinical implications of therapy.

## 2. Immunogenic Features of TNBC

### 2.1. Immune Infiltration

The tumor microenvironment is mainly composed of immune cells, stromal cells, blood vessels, and extracellular matrix [10]. This tumor microenvironment varies not only between different cancer types but also in the same cancer type. Immune cells of the microenvironment are commonly referred to as TILs. TILs may be involved in cancer pathogenesis, knowing that inflammation is one of the hallmarks of cancer, and can also play a therapeutic role depending on their subtypes [11]. Higher numbers of immune cells are linked to a better prognosis, and specific immune cell subtypes, including NK cells, cytotoxic T-cells, and B cells, can suppress the growth of cancer. On the other hand, some immune cell subtypes, such as FOXP3+ Tregs, encourage the formation of tumors. The transcription factor FOXP3, known as the most specific marker of Tregs, plays a crucial role in the development and function of Tregs. FOXP3 is constitutively expressed in the nucleus of human Tregs. The role of FOXP3+ Tregs and CD8+ T-cells in different stages and subtypes of BC is yet to be fully defined. The prognostic importance of tumor-associated FOXP3+ regulatory T-cells (Tregs) and CD8+ cytotoxic T lymphocytes (CTLs) in invasive BC is being investigated. FOXP3+ Tregs, initially identified by their CD4+ CD25+ phenotype, are believed to be a key barrier to antitumor immunity and a challenge for immunotherapy [12].

Numerous studies have demonstrated a correlation between pathological complete response (pCR) and neoadjuvant chemotherapy in BC, either through changes in TIL score or the percentage of a particular subset of T-cells. For instance, a better prognosis was linked to an immunologic profile that combined the absence of inhibitory FOXP3 cells with a high concentration of CD8+ T cytotoxic cells [2, 3]. In general, TILs are strong indicators of tumor immunogenicity, and TNBCs exhibit more abundant lymphoid cell infiltrates than other subtypes of BCs. In a series of 256 BC nodules biopsied by fine-needle aspiration cytology, where 82 were TNBC and 174 were non-TNBC, 55% of the TNBC showed an infiltration by many lymphocytes, whereas this infiltration was present in 8.6% of non-TNBC [13]. In another study also, 50% of TNBC showed an abundant lymphocytic infiltration [14].

It is also worth mentioning that one of the critical factors influencing T-cell reactivity and treatment outcomes in TNBC is the expression of MHC Class I molecules. A study led by Messaoudene et al. [15] revealed significant downregulation of MHC Class I expression in metastatic lymph nodes compared to primary BC tumors, particularly in TNBC. This downregulation can lead to decreased T-cell recognition and response against tumor cells. Additionally, the study identified genetic defects in *β*2-microglobulin (B2M), which is associated with MHC Class I, predominantly in metastatic cohorts of TNBC. These genetic alterations may contribute to the loss of MHC Class I and further impair the immune response. Furthermore, tumors with evidence of MHC Class I loss of heterozygosity (HLA LOH) before treatment were significantly less likely to achieve a pCR to neoadjuvant chemotherapy, indicating that MHC Class I expression status may serve as a predictive biomarker for treatment response in TNBC. Consequently, compromised MHC Class I expression in TNBC, especially in metastatic lymph nodes, may contribute to immune evasion and poorer treatment responses. Understanding these dynamics is crucial for developing effective immunotherapies for patients with TNBC.

### 2.2. Tumor Mutational Burden (TMB)

TMB is defined as the number of somatic mutations per megabase (mut/MB) of DNA using whole exome or gene panel sequencing. A high TMB is associated with more neoantigen formation and increased T-cell infiltration [16]. Recent clinical studies have associated high TMB with a better clinical outcome and increased survival after immunotherapy [17]. Regarding TMB in BC, data is still limited. However, TNBCs were shown to have a higher TMB than other subtypes and were associated with a high degree of chromosome instability and mutations [18]. Barroso-Sousa et al. calculated the TMB from whole exome sequencing (WES) or gene panel sequencing data from 3969 individuals with primary or metastatic BC from six different publicly available genomic studies. Their results showed that TNBC had significantly higher median TMB (1.8 mut/Mb) compared with HR-positive (1.1 mut/Mb) or HER2-positive cancers (1.3 mut/Mb) [19]. In KEYNOTE-119, Winer et al. evaluated the association of TMB with the efficacy of pembrolizumab monotherapy versus chemotherapy in patients with previously treated metastatic TNBC, they found that 26 out of 253 patients (10.3%) had high TMB (TMB ≥ 10 mut/Mb)) [20]. In another study including 62 females with mTNBC, the median TMB was six mut/Mb, and 12 (18%) patients were classified as having high TMB. The most commonly mutated genes were TP53 (82%), BRCA1 (16%), and ATM (13%), and the ORR was higher for patients with high TMB (58%) versus those with low TMB (30%; *p* = 0.09) [21, 22].

### 2.3. PD-L1 Expression

The expression of PD-L1/PD-1 in TNBC has garnered significant attention due to its potential implications for immunotherapy. Several studies have investigated the role of PD-L1 in TNBC, shedding light on its prevalence, clinical significance, and therapeutic implications. For example, a study by Mittendorf et al. highlighted the heterogeneity of PD-L1 expression within TNBC. They found PD-L1 to be expressed in 20% of TNBC cases. This expression was in part regulated by the loss of the phosphatase and tensin homolog (PTEN), hinting that agents targeting the PI3K pathway may increase the antitumor adaptive immune responses [23]. In a separate investigation conducted by Roesler et al. [24] in 2015, they found that there was a genetic amplification occurring at 9p24.1, specifically affecting the genes JAK2, PD-L1, and PD-L2, known as the PDJ amplicon. This amplification was observed in TNBC but not ER+ and HER2+ BC. Additionally, the study revealed a significant correlation between the presence of the PDJ amplicon and elevated PD-L1 expression in TNBC [25]. PD-1 expression was also influenced by genetic factors. In a recent study, Nolan et al. demonstrated that TNBC with BRCA1 mutations displayed higher somatic mutational load and higher expression of PD-1 and CTLA4 when compared to TNBC without BRCA1 mutations [26]. Another important point to raise while looking at PD-L1 expression in TNBC is the role of tumor-associated macrophages (TAMs) in modulating the expression and functions of PD-1/PD-L1 in TNBC. As shown by Zhang et al. in 2017, TAMs have the capacity to stimulate PD-L1 expression by releasing IFN-*γ* through the JAK/STAT3 and the PI3K/AKT signaling pathways [27]. In this context, a proposed predictor of clinical response to PD-1 blockade is the IFN-*γ*-related mRNA profile [28].

## 3. Role of Immunotherapy

The fundamental principle of immunotherapy is the activation of the immune system using immune modulators and tumor-specific antibodies, or through passive immunization with cancer vaccines. Immune checkpoints are immune system regulatory sites that have the power to stimulate or repress the immunological response. For instance, the immune response is negatively regulated by PD-1 and CTLA-4, which also blocks the antitumor response [29]. Historically, treatment options for TNBC were limited to cytotoxic chemotherapy. However, the role of immunotherapy in treating TNBC is rapidly evolving and is now accepted as part of neoadjuvant and adjuvant therapy in early-stage TNBC and as a first-line treatment for metastatic TNBC. Here, we reveal clinical trials that evaluated the efficacy of immunotherapy in both settings.

### 3.1. Immunotherapy in Early-Stage TNBC

In KEYNOTE-522, patients with early-stage TNBC were randomly assigned to receive pembrolizumab or a placebo for eight cycles as part of a neoadjuvant regimen that included paclitaxel and carboplatin, followed by treatment with anthracycline and cyclophosphamide. The pCR rate in the pembrolizumab chemotherapy group was 13.6% higher than the placebo chemotherapy group. Furthermore, the predicted event-free survival at 36 months increased from 76.8% (95% CI, 72.2–80.7) in the placebo chemotherapy group to 84.5% (95% CI, 81.7–86.9) in the pembrolizumab chemotherapy group [30]. In the GeparNuevo trial, durvalumab, a PD-L1 inhibitor, was added to neoadjuvant chemotherapy. This resulted in a considerable but nonsignificant rise in the pCR rate by an absolute 9% (*p* = 0.287). However, additional data from GeparNuevo showed significant improvements in the 3-year invasive disease-free survival (84.9% vs. 76.9%, HR 0.54, *p* = 0.0559) and 3-year overall survival (OS) with durvalumab versus placebo (95.1% vs. 83.1%, HR 0.26, *p* = 0.076) [31]. Similarly, in the NeoTRIP study, researchers assessed carboplatin and nab-paclitaxel chemotherapy with or without atezolizumab in the neoadjuvant setting and revealed no significant improvement in pCR rate after treatment with atezolizumab [32]. Another randomized double-blind clinical trial, IMpassion031, compared the effects of neoadjuvant atezolizumab in combination with sequential nab-paclitaxel and anthracycline-based chemotherapy versus placebo and chemotherapy in patients with early TNBC. pCR was found in 58% of the patients in the first group and 41% in the placebo group, with a rate difference of 17% (95% CI 6–27; one-sided *p* = 0.0044). In the PD-L1-positive population, pCR was documented in 69% of patients in the first group and in 49% in the second group, with a rate difference of 20% (95% CI 4–35; one-sided *p* = 0.021) [33].  Table 1 details the results of different studies adding immunotherapy to neoadjuvant chemotherapy in early-stage TNBC.

### 3.2. Immunotherapy in Metastatic TNBC

IMpassion130 is a Phase 3 study assessing the efficacy of atezolizumab in the metastatic setting with nab-paclitaxel. Four hundred fifty-one patients were randomized, and progressing free survival (PFS) and OS were the main outcomes. PFS in the atezolizumab group was 7.2 months compared to 5.5 months in the placebo group (HR for progression or death, 0.80; 95% [CI], 0.69–0.92; *p* = 0.002), and OS was 21.3 months in the atezolizumab group compared to 17.6 months in the placebo group (HR for death, 0.84; 95% CI, 0.69–1.02; *p* = 0.08) [34].

KEYNOTE-355, another Phase 3 study, evaluated pembrolizumab with chemotherapy versus a placebo arm in untreated locally recurrent inoperable or metastatic TNBC. One thousand three hundred seventy-two patients were randomly assigned in a 2:1 ratio to each group. The median PFS for patients with a combined positive score (CPS) ≥ 10 was 9.7 months for pembrolizumab chemotherapy and 5.6 months for placebo chemotherapy (HR for progression or death, 0.65, 95% [CI] 0.49–0.86; one-sided *p* = 0.0012). For patients with a CPS of one or more, the median progression-free survival was 7.6 and 5.6 months (HR, 0.74, 0.61–0.90; one-sided *p* = 0.0014), while for the intention-to-treat population, it was 7.5 and 5.6 months (HR, 0.82, 0.69–0.97) [35]. The OS in the pembrolizumab chemotherapy group was 17.2 months versus 15.5 in the placebo chemotherapy group. However, for the OS for PD-L1-positive patients with CPS ≥ 10, the median OS was 23 months in the pembrolizumab with chemotherapy group versus 16.1 months in the placebo chemotherapy group (HR for death, 0.73; 95% [CI], 0.55–0.95; two-sided *p* = 0.0185) [36]. This shows that the effects of pembrolizumab treatment were enhanced by PD-L1 enrichment. This study led to the approval of pembrolizumab in metastatic TNBC in patients with PD-L1 CPS ≥ 10.

TORCHLIGHT, another double-blind, randomized clinical trial compared the effects of toripalimab to placebo in combination with nab-paclitaxel in patients with metastatic or recurrent TNBC. After a follow-up of 14 months, a statistically significant improvement in PFS in the first group was found in the PD-L1 positive subgroup (mPFS 8.4 vs. 5.6 months; HR = 0.653, 95% CI 0.470–0.906, *p* = 0.0102) and in the ITT group (mPFS 8.4 vs. 6.9 months, HR = 0.773, 95% CI 0.602–0.994). OS was also improved in the PD-L1 positive group (mOS 32.8 vs. 19.5 months; HR = 0.615, 95% CI 0.414–0.914) and in the ITT group (mOS 33.1 vs. 23.5 months; HR = 0.691, 95% CI 0.513–0.932) [37].

## 4. Challenges and Future Directions

### 4.1. TILs

TILs may be an antitumor immune response in the tumor microenvironment and can be used as a prognosis marker [38]. A study that included 481 patients with TNBC showed that in tumors with 80% TILs in the stromal compartments, the prognosis was better. The increase of TILs in the stromal environment was associated with a reduced risk of distant recurrence and death, making TILs in TNBC an independent prognostic marker of disease-free survival and OS [39]. Another study assessed neoadjuvant chemotherapy response in TNBC depending on the TILs quantity. TIL quantity was intermediate if the lymphocyte proportion was between 11% and 59% of the total immune cells in the stromal tissue within the tumor and high if the lymphocyte proportion was above 60%. After receiving neoadjuvant chemotherapy, pCR was observed in 31% of patients with low TILs and intermediate TILs, whereas 50% pCR in patients with high TILs [40]. High TILs in TNBC, mainly T-CD8+ [41] and plasma cell infiltration [42], seem to be associated with a better prognosis than low TILs and predict a better response to neoadjuvant and adjuvant chemotherapy [43].

### 4.2. Poly ADP-Ribose Polymerase (PARP) Inhibitors

10%–20% of TNBC patients carry a BRCA1/2 mutation, and approximately 70% of BRCA1/2 mutated BC are TNBC [44]. PARP inhibitors, olaparib and talazoparib, have been granted FDA approval for the treatment of BRCA-associated HER2-negative metastatic BC based on the results from the Phase III Olympiad and EMBRACA trials [45]. In the Olympiad study, 302 metastatic BC patients, among which 49.8% were TNBC, were randomized in a 2:1 ratio to receive either olaparib 300 mg as a monotherapy or chemotherapy. Results showed an increase in PFS (7.0 vs. 4.2 months; HR: 0.58; 95% CI: 0.43–0.80; *p* < 0.001) and ORR (59.8 vs. 29.8%) in the olaparib group versus the chemotherapy group with fewer Grade 3 side effects. Similarly, EMBARCA, a Phase III trial comparing talazoparib at a dose of 1.0 mg daily to chemotherapy with 40% of patients having TNBC, showed a median PFS higher in the talazoparib group than the chemotherapy group (8.6 months; 95% CI, 7.2–9.3 vs. 5.6 months; CI, 4.2–6.7; *p* < 0.001). However, both studies did not find a significant increase in OS. This might be explained by the fact that the response to PARP inhibitors has not been durable. Despite the fact that the role of PARPib as a therapeutic milestone is now confirmed in the management of BRCA-mutant TNBC, approximately 50% of patients progressed during treatment [46]. To overcome PARPib resistance, new combination strategies are being developed. For instance, PARPib is known to increase T-cell infiltration inside the tumor. This mechanism is mediated by interferons released from tumor cells with abnormal, unrepaired DNA in their cytoplasm. PARP inhibition also increases tumor neoantigen expression and upregulation of PD-L1 expression [47]. The association of immune checkpoint inhibitors (ICI) to PARPib could improve response to treatment and increase antitumor immunity.

A Phase 1/2 trial (MEDIOLA) of durvalumab and olaparib in solid cancer enrolled 34 patients with germline BRCA1 and/or BRCA2 mutated HER2-negative and metastatic BC. Patients received 300 mg olaparib tablets twice daily for 4 weeks, then a combination of olaparib 300 mg twice daily and durvalumab 1.5 g every 4 weeks until disease progression. After 12 weeks, the disease control rate (DCR) was 24/30 (80%) and 15/30 (50%) after 28 weeks. The PFS was 8.2 months for the overall cohort and a PFS of 9.9 for patients who had zero prior lines of treatment [48].

For early BC, recommendations for systemic therapy to date have been mostly independent of BRCA1/2 status. The standard treatment for early (Stages II–III) TNBC includes pembrolizumab, based on the positive results of the KEYNOTE-522 trial. However, no patients in the KEYNOTE-522 study received adjuvant capecitabine or olaparib, which were not considered conventional treatments at the time of trial design, and regardless of the pCR status, all patients received pembrolizumab in the adjuvant context [30]. The Phase 3 OlympiA study examined the use of adjuvant olaparib in patients with high-risk, HER2-negative BC with a genetic BRCA1/2 mutation who had undergone either adjuvant or neoadjuvant chemotherapy. With a median follow-up of 3.5 years, the analysis found that olaparib significantly improved OS (HR 0.68; 98.5% [CI] 0.47–0.97; *p* = 0.009), with an absolute 3.4% benefit at 4 years. Furthermore, 4 years IDFS (invasive disease-free survival) was improved in adjuvant olaparib 82.7% and 75.4% in the placebo arm (difference 7.3%, 95% CI 3.0%–11.5%). Four-year distant disease-free survival (DDFS) was 87% in the adjuvant olaparib group compared to 79% in the placebo group (difference 7.4%, 95% CI 3.6%–11.3%) [49]. However, after reviewing these trials, one question remains unanswered: What is the best option between olaparib and immunotherapy in BRCA patients with residual disease after neoadjuvant treatment? To answer the question, we need to design trials taking into consideration BRCA status and immunotherapy usage in the neoadjuvant setting.

Next-generation PARP inhibitors represent a promising advancement in cancer therapy. AZD5305 is a next-generation PARP inhibitor designed to selectively inhibit and trap PARP1, achieving significant antitumor activity while minimizing effects on PARP2, which is crucial for the survival of hematopoietic/stem progenitor cells. In vitro studies demonstrated that AZD5305 effectively inhibits PARylation and exhibits potent antiproliferative activity in DNA repair-deficient cells. These findings support the hypothesis that targeting only PARP1 can provide therapeutic benefits without the risks of hematotoxicity, positioning AZD5305 as a promising candidate currently in Phase I trials [50].

Another PARP inhibitor is AZD9574, a blood–brain barrier (BBB) penetrant selective inhibitor of PARP1, evaluated for its properties and activity both alone and in combination with temozolomide (TMZ) in preclinical models. The results showed that AZD9574 exhibited PARP1 selectivity and effective BBB penetration, demonstrating potent efficacy as a single agent in models with homologous recombination repair deficiency. Notably, in an orthotopic glioma model with O6-methylguanine-DNA methyltransferase (MGMT) methylation, the combination of AZD9574 with TMZ significantly extended survival compared to TMZ alone. The unique combination of PARP1 selectivity, trapping profile, and high CNS penetration positions AZD9574 as a best-in-class PARP inhibitor for treating primary and secondary brain tumors.

### 4.3. Antibody-Drug Conjugates (ADCs)

With TNBC lacking ER, PR, and HER2, trophoblast cell-surface antigen (Trop-2) is a promising biomarker in TNBC as it is often upregulated in TNBC. Its overexpression is associated with increased tumor aggressiveness, invasiveness, and resistance to treatment, making it a potential target for therapeutic intervention [51]. ADCs utilize target-specific antibodies as vehicles to deliver a potent cytotoxic to tumor cells while sparing healthy cells, thus limiting toxicity. The high expression of Trop-2 in TNBC cells makes it a suitable candidate for targeted therapies, such as ADCs like sacituzumab govitecan (SG), which exploit Trop-2 as a specific binding site to deliver cytotoxic agents directly to cancer cells [52].

SG, now employed clinically for treating TNBC, is a humanized monoclonal IgG1 kappa antibody targeting Trop-2. The antibody is coupled to SN-38, an active metabolite of the anticancer drug irinotecan, through a hydrolysable CL2A linker. Upon binding to Trop-2, SG releases SN-38 into the tumor cell through linker hydrolysis, facilitated by a pH-dependent cleavage site. The membrane-permeable nature of SN-38 allows for potential antitumor effects on nearby non-Trop-2-expressing tumor cells. SG triggers the intrinsic pathway of cell death in tumor cells, involving caspase activation and PARP cleavage. Moreover, it possesses an enhanced drug-to-antibody ratio of 7.6. These features highlight the potency of SG in targeting TNBC [53].

Many studies evaluated the safety and efficacy of SG in TNBC patients. For example, the ASCENT Phase 3 clinical trial assessed SG versus single-agent chemotherapy in patients with relapsed or refractory metastatic TNBC. Among 468 patients without brain metastases, SG demonstrated significantly prolonged progression-free survival (5.6 months) compared to chemotherapy (1.7 months), along with a significantly extended OS (12.1 months vs. 6.7 months). Objective response rates were markedly higher with SG (35% vs. 5%). Subgroup analyses confirmed the consistent benefit across various patient characteristics. While myelosuppression and diarrhea were more frequent with SG, the safety profile allowed for long-duration administration (median: 4.4 months). Serious adverse events were not frequent. The results suggest SG as a promising therapeutic option for metastatic TNBC, offering improved efficacy compared to standard chemotherapy with manageable adverse events [32, 33].

### 4.4. AR Inhibitors

The role of androgens in the development of BC has been very contrasting. One of the theories is that a loss of equilibrium between estrogens and androgens can increase the risk of the development of BC. Knowing that androgens have antiproliferative properties during puberty and oppose estrogens, they are also estrogen precursors, so an excess in androgen can cause an excess in estrogen and, in consequence, an overstimulation of cell proliferation and an increased risk of BC [54]. This mechanism has mainly been implicated in the genesis of ER+ BC [55] but also in ER− HER2+, where the AR positivity has been associated with overexpression of HER2 [56]. 12%–50% of TNBC express the AR as well [57]. A subtype of TNBC in the Lehmann molecular classification relies on the AR positivity, the LAR TNBC [7]. This subtype has been shown to be less proliferative and less sensitive to chemotherapy than other subtypes [58] and is associated with a lower clinical stage and a lower histologic grade [59]. Furthermore, AR negativity has been linked to an increased risk of recurrence and metastasis [60]. AR positivity in TNBC has also been associated with better survival than other TNBC subtypes [61]. This suggests that AR can be used as a therapeutic target in the treatment of BC and as an alternative to traditional cancer therapies like chemotherapy.

Indeed, androgen-targeted therapies, like enzalutamide, a nonsteroidal antiandrogen drug, have been shown to have a benefit in ER+ and ER− AR+ BCs, either alone or in combination with other therapies [62]. Numerous clinical trials are ongoing to further evaluate the response of AR+ TNBC to androgen-targeted therapies. A study where AR+ TNBC patients were treated with bicalutamide, a nonsteroidal antiandrogen, has shown a clinical benefit of 19% and a median progression-free survival of 12 weeks [63]. An ongoing study (Phase II) on AR+ TNBC is showing a clinical benefit of 20% for bicalutamide and 28%–33% for enzalutamide [64]. Another ongoing study is evaluating the benefit of combining enzalutamide with paclitaxel in early AR+ TNBC [57].

Furthermore, AR activity has been linked to increased DNA damage repair, which can be another route of action for androgen-targeted therapies [65]. The targeting of AR in association with PARP inhibition modulates DNA damage repair, leading to increased antitumor activity in AR+ TNBC [66]. A benefit has also been shown in a combination of AR targeting with radiotherapy in AR+ TNBC, where AR modulation was used not only as a targeted therapy but also as a radio-sensitizing drug, leading to an amplified response of the tumor to radiotherapy [67].

## 5. Conclusion

Different factors come into play with TNBC exhibiting significant heterogeneity across clinical and histopathological levels, with genomic instability and increased mutation rates contributing to its aggressiveness [4]. Patients with TNBC often undergo various therapies aimed at targeting this aggressiveness. Chemotherapy is a common treatment but can lead to side effects such as fatigue, nausea, hair loss, and increased susceptibility to infections due to its impact on healthy cells. Radiation therapy, while effective for local tumor control, may cause skin irritation, fatigue, and, in rare cases, damage to nearby organs like the lungs or heart. Emerging treatments, including immunotherapy and targeted therapies, offer new hope but also come with associated risks. For instance, immunotherapy can lead to immune-related side effects such as pneumonitis. Targeted therapies, such as PARP inhibitors, may cause nausea, anemia, or low platelet counts, necessitating careful management. Moreover, there is growing evidence that TILs in TNBC have prognostic value and are associated with clinical outcomes and improved survival [5]. While evading antitumor immunity is a hallmark of the progression of cancer [6], immunotherapy studies have focused on the role of the PD-1/PD-L1 pathway in maintaining immunosuppression in the tumor microenvironment. Blockade of the PD-1/PD-L1 axis has emerged as a promising therapeutic option to enhance antitumor immunity and is actively being investigated in TNBC, with encouraging results influencing therapy both in the early stages as well as in the metastatic setting. Furthermore, immunotherapy, ADCs, and PARP inhibitors, among others, represent novel therapeutic options with concrete clinical results.

## Figures and Tables

**Table 1:** Summary of studies adding immunotherapy to neoadjuvant chemotherapy in early-stage TNBC.

**Study**	**Treatment**	**Pathological complete response rate**	**p**	**Event-free survival/overall survival**
GeparNuevo Phase 2 trial 174 patients Stages I–III	Paclitaxel/AC ± Durvalumab	44.2 vs. 53.4% OR: 1.53 (95% CI 0.82–2.84)	0.182	3-year iDFS 77.2 vs. 85.6% HR = 0.48 (95% CI 0.24–0.97; *p* = 0.0398) 3-year OS 83.5 vs. 95.2% HR = 0.24 (95% CI 0.08–0.72; *p* = 0.0108)
KEYNOTE-522 Phase 3 trial 1174 patients Stages II–III	Paclitaxel/carboplatin Then AC/EC ± Pembrolizumab	51.2 vs. 63.8% *p* < 0.001 for 604 patients	< 0.001	3-year EFS 76.8 vs. 84.5% HR = 0.63 (95% CI 0.48–0.88; *p* = 0.0003) OS Not mature
NeoTRIP Phase 3 trial 280 patients Stages II–III	Paclitaxel/carboplatin ± Atezolizumab	44.4 vs. 48.6% OR: 1.18 (95% CI 0.74–1.89)	0.48	5-year EFS 74.9 vs. 70.6% HR = 1.076 *p* = 0.76
IMPassion031 Phase 3 trial 333 patients Stages II–III	Nab-paclitaxel Then AC ± Atezolizumab	41.1 vs. 57.6%	0.0044	Not reported

## Data Availability

No datasets were generated or analyzed during the current study.
